# An exploration of rollover protective structures (ROPS) rebate program media coverage: strategies for implementation and sustainment

**DOI:** 10.1186/s12889-019-7586-3

**Published:** 2019-09-11

**Authors:** Pamela J. Tinc, Julie A. Sorensen, Lars Weinehall, Kristina Lindvall

**Affiliations:** 1 Northeast Center for Occupational Health and Safety: Agriculture, Forestry, and Fishing, 1 Atwell Road, Cooperstown, NY 13326 USA; 2 Department of Epidemiology and Global Health, Umeå University, 901 87 Umeå, Sweden

**Keywords:** Rollover protection, Media advocacy, Implementation, Discourse analysis

## Abstract

**Background:**

Media advocacy plays an important role in public health initiatives, as it can provide vital information to target populations, policy makers, or other relevant stakeholders. Unfortunately, little is currently known about the use of media advocacy to promote occupational safety and health programs. This study explores media coverage related to the Rollover Protection Structure (ROPS) Rebate Programs, which were designed to encourage the use of rollover protection on agricultural tractors, thus reducing the risk of tractor overturn fatalities. The Program’s portrayal in the media, as well as the role that the media has played in implementing and sustaining these Programs.

**Methods:**

Media articles pertaining to any of the state-based or National ROPS Rebate Programs and published between November 1, 2006 and October 31, 2018 were included for review. Discourse analysis was used to understand the messages portrayed by the media and how those messages shaped the outcomes of the ROPS Rebate Programs.

**Results:**

During the study period, 212 unique articles were published about the ROPS Rebate Programs. While these articles all portrayed the ROPS Rebate Programs in a largely positive light, they were used at different stages, from pre-implementation through sustainment of the ROPS Rebate Programs, and to different extents.

**Conclusions:**

Media articles have played an important role in implementing and sustaining the ROPS Rebate Programs. Based on the results of this study, more robust and continuous media coverage are important for the longevity and success of public health programs.

## Background

As it relates to public health, media can both help and hinder efforts. Similarly, media can both influence and be influenced by readers at all levels from policy and decision makers to the general public. As such, media advocacy efforts, which can be defined as, “the strategic use of mass media to support community organizing and advance healthy public policy [1],” have become important components of many public health interventions, both to negate inaccurate reports and promote efficacious and effective public health efforts [1]. Largely, these campaigns have focused on more traditional areas of public health, such as smoking cessation [1–4]; however, some reports have recommended media advocacy as a strategy for promoting occupational safety initiatives as well [5]. Unfortunately, published media advocacy evaluations focused on occupational safety could not be identified. Thus, there is no published evidence about what components of media advocacy are most effective in encouraging the spread of evidence-based occupational safety interventions.

To help fill this gap, this study seeks to explore and understand a series of media advocacy campaigns surrounding an agricultural safety intervention first developed in New York State and then expanded to additional states and eventually nationally.

### The Rollover Protective Structure (ROPS) Rebate Program

#### Development and expansion of the ROPS Rebate Program

Given that the average working adult spends more than a quarter of their lives at work [6, 7], occupational safety and health is a key public health priority. This is especially true in occupations such as US agriculture, where work hours tend to be longer and the fatality rate is 6.8 times higher than the all worker fatality rate [8]. In the US, tractor overturn fatalities are the leading cause of agricultural deaths, despite available technologies to prevent such tragedies. Rollover protective structures (ROPS) provide a protective zone around tractor operators so that in the event of an overturn, the operator is protected. When used with a seatbelt, ROPS are 99% effective in preventing deaths and serious injuries; without seatbelts, they are still effective in reducing deaths [9]. ROPS are standard equipment on tractors built since 1985; however, many tractors in use today are older, and thus need to be retrofitted: a process which is both time-consuming and expensive [10, 11].

In 2006, the first ROPS Rebate Program was implemented in New York State to reduce farmers’ barriers to installing ROPS on their older tractors. The Program consists of three primary components: 1) a social marketing campaign aimed at raising awareness of tractor overturn fatalities and the Program, 2) a hotline to assist farmers with identifying the correct ROPS kit for their tractor, and 3) a 70% rebate toward the cost of purchasing and installing a ROPS kit [12]. These components, together, address the primary barriers farmers face in retrofitting: denial of personal risk, time, and finances [10–12].

Due to the success of the New York Program, the ROPS Rebate Program model was replicated to varying extents in Vermont, New Hampshire, Pennsylvania, Massachusetts, Wisconsin, and Minnesota between 2010 and 2016.

**Fig. 1:**
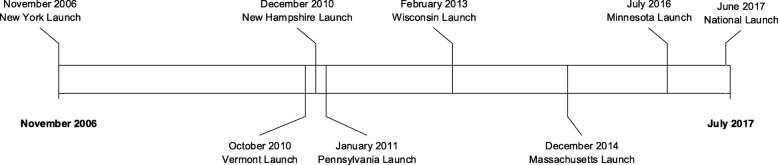
Timeline of ROPS Rebate Program implementation in seven US states and nationally

In addition to individual state Programs, the National ROPS Rebate Program was launched in 2017 [13]. The National ROPS Rebate Program serves two purposes: 1) to support and facilitate individual state Programs, and 2) to obtain national-level funding to supplement state-allocated funds. Figure 1 shows a timeline demonstrating the expansion of the ROPS Rebate Programs.

Though all ROPS Rebate Programs currently operate under the umbrella of the National ROPS Rebate Program, they are referred to as individual ROPS Rebate Programs in this manuscript. This is in order to highlight the role that media has played in each Program, and how differences in these media strategies could have contributed to differing outcomes.

#### Success of the ROPS rebate programs

The short-term outcomes presented in this study are the amount of funding dedicated to ROPS Rebate Programs, as well as the number of retrofits through the Programs.

New York, which was the first state to launch a ROPS Rebate Program, has dedicated the largest amount of funding for rebates, and has thus retrofitted the greatest number of tractors (1765 in 12.5 years). Rebate funding in New York is provided by the New York State Department of Agriculture and Markets.

Both Vermont and Pennsylvania fully implemented the three core components of the ROPS Rebate Program in 2010 and 2011; however, both states relied on private donations to fund rebates. Unfortunately, this resulted in the quick depletion of rebate funding and the development of waitlists by 2012. These waitlists continue today. The New Hampshire Program, which is funded through private donations, and the Massachusetts Program, which is funded through the State Department of Agricultural Resources, also implemented all aspects of the ROPS Rebate Program; however, with less emphasis on the social marketing campaign. As such, demand for the Programs is limited, and rebate funding has been sufficient to meet those demands.

Both Wisconsin and Minnesota have ample funding, and are able to utilize all aspects of the ROPS Rebate Program. However, while Minnesota’s rebate funding is provided by the state legislature, rebate funding in Wisconsin is obtained through an annual fundraiser. In recent years, organizers of this fundraiser have discussed redirecting the funds to another agricultural safety and health program, thus challenging the sustainability of funding. In response, Wisconsin Program staff have begun efforts to obtain other sources of funding.

Finally, the National ROPS Rebate Program has had some success in obtaining rebate funding ($33,771) and retrofitting tractors (13 since 2017). However, a more important short-term outcome in this case is the preservation of funding for administrative tasks. This includes the cost of facilitating the ROPS Rebate Programs (hotline, website, rebate fund tracking) and working to procure funding in under-served areas. At the time of this study, this funding has been provided continuously since the Program launched.

Long-term outcomes of the ROPS Rebate Programs include the number of lives saved, as well as resulting cost savings. Because these outcomes require a great deal of data over a longer period of time, they have so far only been assessed relative to the New York ROPS Rebate Program. Recently published research demonstrates that this Program not only save lives, but are fiscally advantageous, as the cost to administer them is markedly less than the cost of addressing overturn fatalities and injuries once they have occurred [14]. Despite these successes, it has been difficult to administer new and existing Programs because of funding challenges. Given the evidence demonstrating the value of these programs, it is important to understand the challenges in achieving widespread adoption for a proven solution to one of the most prominent farm injury issues.

#### Media and the ROPS Rebate Programs

As part of the implementation and sustainability strategies, media has been used to varying extents across all ROPS Rebate Programs to share information, promote the Programs, and gain political and public support. Though used frequently to promote the Programs, no pre-planned media advocacy strategies existed for any of ROPS Rebate Programs. Instead, media was used as opportunities arose in the various settings (for example, when new funding became available or retrofitting milestones were reached). Despite these efforts, in interviews with stakeholders involved in the implementation of the National ROPS Rebate Program conducted in 2017 and 2018 (Tinc et al., forthcoming), the individuals interviewed were generally unaware of media or related political commentary surrounding the National ROPS Rebate Program, thus leaving a major gap in fully understanding the implementation efforts. This study aims to fills this gap by assessing 1) how the ROPS Rebate Programs are portrayed in the media, and 2) how media are used to advance the ROPS Rebate Programs.

## Methods

### Study framework

This study used a discourse analysis for print media approach, which begins with collecting media publications relevant to the topic. In doing so, specific key words are used to monitor media sources for real-time updates and retrospectively search online databases to identify published media reports.

Discourse analysis assumes that language both shapes, and is shaped by the real world [15–17]. In analyzing print media, discourse analysis can be helpful in demonstrating how media portrayals of current events can shape perceptions, and thus the trajectory of those events [17]. This study, which focuses on better understanding how media has shaped the process of implementation and sustainability in the case of the ROPS Rebate Programs, is thus a prime candidate for discourse analysis.

In conducting discourse analysis of print media, three areas of consideration are of particular interest: the text or news story, the process used to develop the news story, and the news story’s relationship to the audience [18].

Textual analysis, which is most common in discourse analysis [18] can be largely discussed in terms of the language used (tone and content) as well as the power structures portrayed through the final text. The implied tone of the language used, based on our cultural understanding of language and key phrases, helps to frame issues in contexts of right versus wrong or good versus bad [17]. As such, two media reports focused on the same event or topic can have differing meanings, and thus outcomes, based on the language that they are presented in. These different meanings may result from the stories that are told, the specific focus of the stories (for example, details of actions versus emotional impact), and the labels that are given to people, events, and ideas [17].

This links to the process used to develop the media report in that those of differing social status or power (relevant to the media report) may be used in varying ways during the development process. In the case of the ROPS Rebate Programs, an array of individuals from varying backgrounds (e.g. politicians, researchers, farmers) are used as sources of information and quoted in media reports.

Finally, the language used in the text as well as the power and societal structures defined help to dictate (or are dictated by) the audience for the particular media report. Together, these can be important tools in shaping or reinforcing the readers’ opinion of the topic, the individuals involved, and the societal hierarchy relevant to the issue [17, 19].

### Data collection

Media reports that related to the ROPS Rebate Program were gathered from collections that Program staff had maintained since its inception in November 2006. These collections were developed based on web-based media alerts set up through Burrelles Luce News Clippings and Google Alerts, as well as visual inspection of printed media publications upon receipt. In order to ensure that all media reports were included, ProQuest Newspapers was searched using the key term “ROPS Rebate Program.” All media reports referencing one of the ROPS Rebate Programs and published before October 31, 2018 were included in this analysis. Media reports were excluded if they only mentioned the Program as part of a list of an organization’s services and did not provide additional detail.

### Data analysis

#### Categorization of media reports

Media reports were first organized based on the date in which they were published, as well as the specific ROPS Rebate Program that was referenced (i.e. an individual state or the National ROPS Rebate Program). Media reports that referenced multiple state Programs, but were published prior to use of the term “National ROPS Rebate Program” were still considered to be related to the National Program. By organizing the media reports in this way, the authors were then able to identify reports that had been replicated either in full or partially. Additionally, this allowed for media discourse to be compared between each of the current ROPS Rebate Programs, as well as states that do not yet have Programs. Analysis of media reports by state was key to the aims of this study, as it allows for assessment of how differences in media strategies can contribute to different outcomes in each case.

#### Discourse analysis of media reports

The lead author, who is heavily involved in the administration of the ROPS Rebate Programs, first inductively coded each media report line-by-line using NVIVO 12 qualitative analysis software [20]. Throughout the coding process, memos were used to maintain notes and ideas related to the data, the analysis, and the context surrounding the media sources. Using these codes and memos, similarities and differences in the portrayal of the ROPS Rebate Programs and the strategies applied were identified by the four authors (two of whom are involved in the day-to-day workings of the Programs, while the others have primarily been involved in Program evaluation). These comparisons take into account the three aspects of media: text, process, and audience, as well as the language and power structures identified in the media reports.

An external reviewer who was unfamiliar with the ROPS Rebate Programs provided written summaries of the media reports relevant to each Program. These summaries included overviews of the target audience, key messages, important players, and media strategies used, but did not include in-depth analysis or latent meanings. While conducting the analysis, the authors used these summaries to ensure that their own viewpoints and those of individuals less connected with the ROPS Rebate Programs were maintained and considered in the analysis. Though there were differences in the analyses conducted by the coder and the reviewer, these differences were not related to content. Instead, these differences related to the emphasis given to certain aspects of the data, which was based on each individuals’ knowledge of the Programs. Thus, the minor discrepancies between the coder and the external reviewer served to balance the two perspectives.

## Results

### Overview of media reports

The earliest identified media report was published in New York on November 20, 2006. During the 12-year period included in this study, a total of 212 unique media reports (i.e. new media reports) were published 357 times (Table 1). Full-text versions of these media reports appeared in a total of 294 times, while abbreviated versions were published 63 times. Seventy-three percent (*n* = 259) of these media reports were published through non-agricultural media sources (such as local newspapers) while the remainder appeared in agricultural-specific sources (for example, trade publications such as *Successful Farming*).

**Table 1:** Articles published by year and program

Year	Program	Unique Media Reports	Full Text Copies	Abbreviated Copies	Agricultural Publications	Non-Agricultural Publications^a^
2006	New York	4	5	3	1	7
2007	New York	5	7	0	1	6
2008	New York	10	17	2	2	17
2009	New York	8	14	7	4	17
2010	New York	2	6	0	2	4
	Vermont	2	2	0	0	2
	New Hampshire	4	4	0	1	3
	Wisconsin	1	1	0	0	1
	National	2	2	0	0	2
2011	New York	20	25	3	5	23
	Vermont	3	3	0	0	3
	Pennsylvania	6	6	0	2	4
	National^b^	3	6	4	6	4
	Other	2	2	1	2	1
2012	New York	13	19	3	6	16
	Vermont	1	1	0	0	1
	Pennsylvania	2	4	1	1	4
	Wisconsin	1	1	0	1	0
	National^b^	1	1	0	1	0
2013	New York	4	6	0	2	4
2014	New York	3	3	3	3	3
	Pennsylvania	1	1	0	0	1
	Wisconsin	3	3	0	3	0
	National	9	13	0	8	5
	Other	1	1	0	0	1
2015	New York	1	8	2	3	7
	Wisconsin	2	2	0	1	1
	Massachusetts	1	1	0	1	0
	Minnesota	1	1	0	0	1
	National	1	1	0	1	0
2016	New York	2	19	7	1	25
	New Hampshire	3	3	0	0	3
	Pennsylvania	1	1	0	0	1
	Wisconsin	5	5	0	4	1
	Minnesota	23	27	12	5	34
	National	2	2	0	2	0
	Other	1	1	0	0	1
2017	New York	6	11	1	1	11
	Wisconsin	5	6	4	7	3
	National	22	23	3	3	23
2018	New York	9	10	2	6	6
	Wisconsin	7	8	2	5	5
	National	8	11	3	6	8
	Other	1	1	0	1	0
	TOTAL	212	294	63	98	259

^a^While the vast majority of non-agricultural publications were targeted at the general public, five media reports were published in targeted non-agricultural media publications, including POLITICO (*n* = 2), NIOSH e-News (*n* = 1), Occupational Safety and Health (*n* = 1), and the Association of Equipment Manufacturers Newsletter (*n* = 1). These five media reports all discussed the National ROPS Rebate Program

^b^The National ROPS Rebate Program was first labeled in 2014. Media reports published prior to this, but discussing all Programs available at the time, were considered to be representative of the National ROPS Rebate Program

Of the media reports published; 99 (51%) referred primarily to the New York ROPS Rebate Program, 67 (18.8%) to the National ROPS Rebate Program, 40 (11.2%) to the Minnesota Program, 32 (9%) to the Wisconsin Program, 13 (3.6%) to the Pennsylvania Program, seven (1.9%) to the New Hampshire Program, six (1.6%) to the Vermont Program, six (1.7%) to states without Programs as of October 31, 2018, and 1 (0.2%) to the Massachusetts Program. The numbers of media reports published about each Program are highlighted in comparison to Program outcomes (rebate funding and retrofits) in Table 2.

**Table 2:** Media reports, rebate funding, and retrofits by ROPS Rebate Program

Program (Year of Launch)	Media Reports	Rebate Funding (in US Dollars)	Retrofits
New York (2006)	99	1,267,179	1698
Vermont (2010)	6	168,639	221
New Hampshire (2010)	7	55,437	76
Pennsylvania (2011)	13	78,315	129
Wisconsin (2013)	32	207,876	239
Massachusetts (2014)	1	25,000	24
Minnesota (2016)	40	442,500	351
National (2017)	67	33,771^a^	13

^a^In addition to rebate funding, the sustainment of administrative funding for the National ROPS Rebate Program can be considered an important outcome

**Fig. 2:**
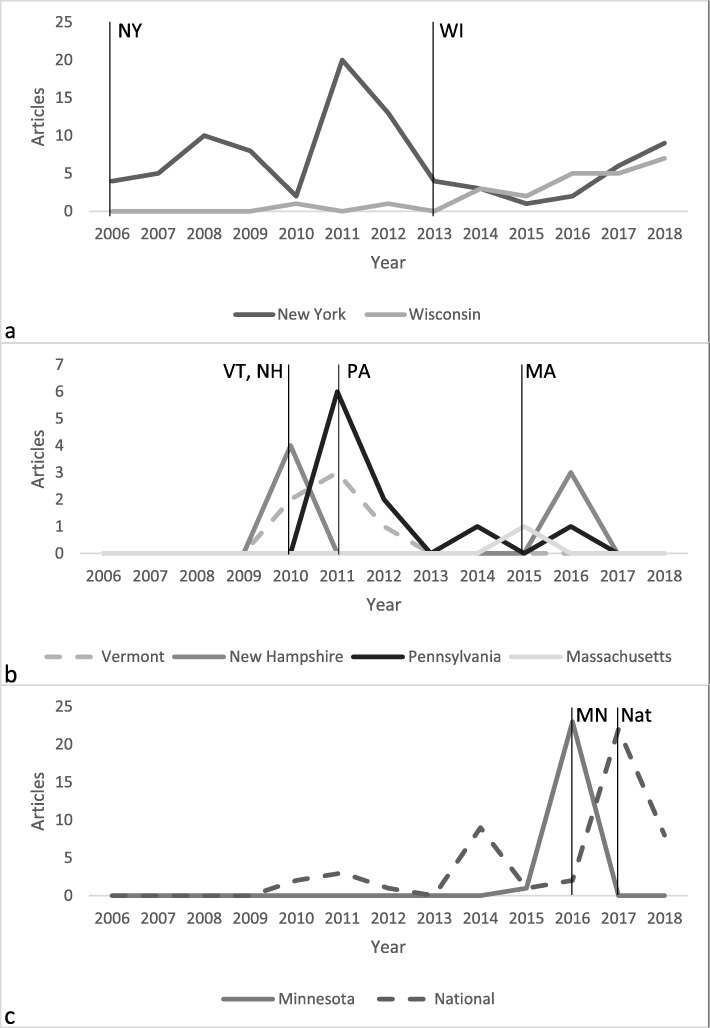
Media reports published by Program and year compared to Program launch dates (indicated by vertical lines). Parts **a**, **b**, and **c** are divided based on when media has been used in relation to the Program launch (continuously after the launch, surrounding the launch only, or primarily before the launch, respectively)

Based on the numbers of unique media reports published compared to the Program launch dates, New York and, to some extent, Wisconsin, have used media extensively since the launch of the initiatives. New Hampshire, Vermont, Pennsylvania, and Massachusetts similarly used media to launch their Programs; however, these efforts were discontinued after a short time. Both the Minnesota and National Programs have used media to introduce the possibility of a Program, and then have continued to varying degrees after their respective launches. These trends are highlighted in Fig. 2.

### Origins of media reports

Based on the media reports review, the origination of these reports (part of the process of developing text) was different across the states. Those published in Vermont, New Hampshire, Pennsylvania, Massachusetts, and Wisconsin primarily stemmed from press releases and announcements provided by Program staff. The New York and National ROPS Rebate Programs’ media involved a mixture of staff-prepared press releases and announcements, political press releases, and organically prepared materials initiated by external journalists and reporters. Finally, on the far end of the spectrum, media reports published in Minnesota were primarily initiated and prepared by journalists and reporters external to the Program.

### Actors portrayed in media reports

Most often, the media reports included in this paper used quotes and input generated from ROPS Rebate Program staff. Examples of these types of media reports could be found for all Programs. Similarly, examples of media reports that featured farmers could also be identified across all Programs. Though farmers were quoted in media reports across all Programs, such examples were most common media reports about the New York ROPS Rebate Program, while the Pennsylvania ROPS Rebate Program media reports incorporated farmer opinions sparingly. In addition to these two groups, legislative officials were portrayed to a great extent in both media reports about the New York and National ROPS Rebate Programs. Legislative officials were also quoted in Minnesota and Wisconsin, but to a much lesser extent. Lastly, other groups, including insurers and equipment dealers were occasionally quoted. Often, the presence of input by these types of organizations were generally tied to prior support for the Programs by the organizations.

### Key audiences of media reports

Two key audiences were identified in these media reports: 1) the farmers who can participate in the ROPS Rebate Programs, and 2) the potential supporters or funders of the Programs, both legislative and non-legislative.

The remainder of the results section is divided into two overarching areas: commonalities across all media reports in this study, and differences between the media reports across ROPS Rebate Programs.

### Common portrayals of the ROPS Rebate Programs

#### ROPS Rebate Programs as expressions of gratitude for farmers

Across the board, media reports related to the various ROPS Rebate Programs tended to highlight the important role that farmers play in their communities and in the United States as a whole. These quotations came from a number of different parties, further highlighting the importance of having farmers in the community. Often, individuals who were quoted speaking of farmers’ importance used this as a way to convey the idea that the Program provides a way to thank farmers for their hard work by providing reduced-cost safety technologies (ROPS).

“Minnesota’s farmers work hard to feed people around the world, and take care of their families and communities. Minnesota’s new Rollover Protective Structures Rebate Program helps to take care of them by defraying the cost of important improvements to aging farm equipment that would otherwise lack modern safety features,” (Government Official; Minnesota Farm Guide, October 1, 2016; Minnesota ROPS Rebate Program).

#### ROPS Rebate Programs are beneficial to farmers and communities

In addition to the ROPS Rebate Programs’ staff using media reports as a method for recognizing farmers and thanking them for their work, the vast societal benefits of the ROPS Rebate Programs were also highlighted in much of the media. The immediate and intended benefit of the Program (to reduce farmer fatalities) was highly praised in many media reports.

“With increasing use of ROPS on tractors we are seeing fewer accidents and fatalities. This is a very effective program for a lot of reasons. Our research data shows the ROPS tractor safety program has made farm work less dangerous,” (National Institute for Occupational Safety and Health Representative; Preston Citizen, June 28, 2017; National ROPS Rebate Program).

In addition, many media reports highlight other benefits of the Program: the long-term impacts on the individual farms, the economic impact of preventing tractor overturn fatalities, and job preservation at agricultural equipment dealerships and ROPS manufacturing organizations.

“What’s more, farmers aren’t just risking their lives when operating tractors without these roll bars, but their entire businesses too,” (Government Official; Empire Farm and Dairy, November 2017; National ROPS Rebate Program).

#### ROPS Rebate Programs are of interest to farmers

Finally, across the majority of media reports, one misconception related to the ROPS Rebate Programs was addressed. This relates to stakeholders belief that farmers’ are not interested in safer work practices or participating in programs such as the NRRP.

“An old friend, a very careful man, was killed in a rollover some years ago. Other neighbors and friends have been thusly injured. On occasion, I get into a situation where I myself am afraid. So there is a clear danger that a rollbar would reduce. Yet, I choose not to install one,” (Economist/Community Member; Pioneer Press, April 2, 2016; Minnesota ROPS Rebate Program).

While occasional media reports have quoted individuals, such as the one above, many more have made an effort to highlight farmers’ need and appreciation for the financial assistance that the ROPS Rebate Programs provide, thus negating opposing arguments.

“You know, most farmers, dairy farmers, they milk in the morning, do field work, have to milk at night, so safety is always a concern and anything we can do to improve safety is what we look for,” (Farmer; Your News Now, April 19, 2011; New York ROPS Rebate Program).

#### Misinformation about the effectiveness and availability of ROPS

In addition to these portrayals, another overarching trend in media reports was that often, incorrect information was provided through the articles. In some cases, this misinformation related to the rebate amount, availability or Programs, or the idea that ROPS are only effective when used with a seatbelt (in reality, ROPS are still effective in reducing deaths if a seatbelt is not worn [9]). In regard to rebate amount and Program availability, the misinformation most often set unnecessary limitations (i.e. lower rebate amounts or indication that fewer states are involved in the Programs).

### Diverse media strategies for advancing the ROPS Rebate Programs

Based on the reviewed media reports, media seem to have been used differently across the various ROPS Rebate Programs. In New York and Minnesota, as well as at the National level, media reports seem to be more closely aligned with principles of media advocacy to support implementation and sustainability of Programs. However, in the remainder of the states, media reports seemed to focus less on advocacy and more on awareness-building. Though both strategies can be seen in almost all efforts, this analysis focuses on the primary use of media within each Program.

#### Media advocacy as a tool for implementing and sustaining the ROPS Rebate Programs

##### Media as a pre-implementation strategy for the Minnesota ROPS Rebate Program

Media in Minnesota was strongest prior to implementation. There, a local reporter prepared a multi-part series on the growing number of fatalities on Minnesota farms, which then sparked the interest of legislators and government officials in the state. In one of the media reports, the journalist stated;

“Lawmakers said they were prompted to act by a 2015 series in the Star Tribune that revealed a disturbing spike in the number of Minnesota farmers killed in work-related accidents,” (Star Tribune, March 23, 2016).

Though ROPS Rebate Program staff were interviewed for this series, the reporter had developed and executed the series based on his own personal interests and without any prior efforts to promote the Program in Minnesota. After the initial media report introducing the ROPS Rebate Program, attention shifted to the growing interest in funding a Program. In several instances, media reports focused on the past success of the ROPS Rebate Programs in other states and legislative interest in mimicking these efforts.

“I thought if other states can do it and feel it’s a worthwhile program, maybe we should try something like that in Minnesota,” (Government Official; St. Cloud Times, April 6, 2016).

The media reports closely followed the legislative discussions surrounding the ROPS Rebate Program with some reports more positive than others. In publishing this information, a few media reports also presented arguments from those who were opposed to the ROPS Rebate Program. In fact, Minnesota was the only state in which explicit opposition to the Program, or parts of it, was depicted in media reports.

“On the other hand, if we want to reduce unnecessary deaths and injuries and as taxpayers are willing to spend an extra $250,000 to do so, would giving it to farmers to buy rollbars for old tractors save the most lives for the money? I think not, but opinions will vary,” (Economist/Community Member; Pioneer Press, April 2, 2016).

Once a bill supporting the ROPS Rebate Program passed in 2016, the media attention shifted. Rather than focusing on the political debate surrounding the Program, most media reports refocused on letting farmers know how the Program works and sharing early successes before media ended just 3 months after the Program launched.

“Since its inception, 150 farmers have been approved for a rollover protective structures…Another 127 farmers have an application pending, some of which are approved and in the process of finding an appropriate rollover protective structure kit,” (ROPS Rebate Program Staff; West Central Tribune, September 29, 2016).

##### Media as a platform for political commitment to implementing and sustaining the New York ROPS Rebate Program

Media reports in New York were used initially to support the launch of the ROPS Rebate Program; however, over time, media has been used as an aide to Program sustainment. Initial media reports related to the New York ROPS Program were largely targeted at farmers and focused on explaining and promoting the Program; however, the strategy used transformed over time. Media reports focused more on farmer stories and evidence of lives saved through the Program as well as arguments in favor of the ROPS Rebate Program as an investment opportunity for farmers.

“I was very fortunate. It prevented (the tractor) from going all the way over and got the tractor upright. If it hadn’t been on it would have probably rolled over and broke my neck,” (Farmer; The Spectator, April 2011).

Though most of the media reports published about the New York ROPS Rebate Program focused on sharing information with farmers, these media reports doubled as a way to engage politicians and other supporters in the public discussion of the Program. About three quarters of these media reports included quotes or were written by state legislators who have supported the Program and assisted in securing state-based rebate funding.

“In the past few years, I have been proud to have secured $1.2 million in funding, including $250,000 in the new state budget, for a program that helps to protect farmers from tractor rollover incidents,” (Government Official; Senator Patty Ritchie’s Weekly Column, May 19, 2017).

In addition, the necessity and importance of legislative support for the Program was often referenced even when legislators were not directly quoted. These quotations served to reinforce the important contribution of political stakeholders.

“I am writing to recognize the leadership role that Senator Catharine Young and the New York Senate Agricultural Committee have assumed to protect the lives and well-being of farmers in our area and throughout New York State,” (ROPS Rebate Program Staff; Wellsville Daily Reporter, February 20, 2008).

##### Politically driven media to sustain support for the National ROPS Rebate Program

Media on the National ROPS Rebate Program varied more in its content and intended target audience than coverage related to individual state Programs. These media reports tended to focus almost entirely on generating support from non-farming stakeholders for the National ROPS Rebate Program, with many including direct asks for support (e.g., in the form of resources or collaborative opportunities).

Almost all of the initial media reports about the National Tractor Safety Coalition referred to the group’s push for a National ROPS Rebate Program modeled off those previously mentioned. In general, these media reports shared basic information and provided additional stakeholders with methods of getting involved.

“Through a series of facilitated exercises over 2 full days, the group created a vision for tractor safety and made organizational commitments to reduce tractor-related deaths in the United States and to promote retrofits of older tractors with roll-over protective structures (ROPS),” (ROPS Rebate Program Staff; NIOSH eNews, June 2014).

After the initial media reports about the Coalition, little was published until June 2017 when the National ROPS Rebate Program was officially launched. However, this launch came at the same time in which cuts to the presidential budget severely threatened continuation of the Program.

“It has been terminated in the past but the cuts are deeper and we are not sure if we are going to survive so I guess the shortest answer to your question is there would be no program. Why should somebody have to put their life at risk to make enough money to make ends meet? It just seems like- it seems unjust,” (ROPS Rebate Program Staff; Utah Public Radio, June 29, 2017).

Having made national headlines, the budget cuts threatening the National ROPS Rebate Program were then addressed through many media reports featuring United States Senator Chuck Schumer, then the Senate Minority Leader. These media reports followed Senator Schumer through a series of press-events in which he addressed the need to maintain funding for the Program and vowed to “[do] everything possible to make sure this program, which puts farmers first, is protected,” (USA Herald, October 11, 2017). In this push, the Senator, and the reporters covering the effort, focused primarily on both the lives saved by the Program, as well as the extensive cost savings seen.

“The work done by organizations like the NEC is exactly the type of work the federal government should be investing in: it’s cost-effective, informed by real industry experts, and helps save farmers’ lives every day,” (Government Official; Evening Tribune, October 10, 2017).

Similar sentiments are echoed throughout the effort to preserve funding, as well as after a press announcement highlighting the Center for Disease Control and Prevention’s commitment to maintain funding for the Program in July 2018.

#### A non-advocacy approach to using media as a promotion tool

##### Using media to announce the ROPS Rebate Programs in Vermont, New Hampshire, Pennsylvania, and Massachusetts

Aside from New York, northeastern states (Vermont, New Hampshire, Pennsylvania, and Massachusetts) focused little attention on sharing Program information through the media. In these states, media reports were primarily limited to the time directly surrounding the launch of each states’ Program. Most of the initial media reports tended to be fact-based and simply described the various aspects of the Program and its importance. In both Pennsylvania and Vermont, media reports were also used to highlight that rebates provided through the Program are from private donations. While well intentioned, these highlights hinted at the potential insufficiency of Program funding and therefore the Program.

“[Vermont], meanwhile, is working to secure more private sponsors for the program: The number of sign-ups is about to eclipse the rebate fund,” (ROPS Rebate Program Staff; Burlington Free Press, October 22, 2010).

##### Media to increase farmer engagement in the Wisconsin ROPS Rebate Program

Media reports in Wisconsin began briefly before the ROPS Rebate Program was launched; however, the vast majority of the reports were published after the start of the Program. In general, these media reports used a three-piece strategy directed at farmers and dictated by Program staff rather than journalists. This strategy involved advertising for the Program by 1) briefly describing the issue of tractor overturns and the purpose of ROPS, 2) announcing the availability of the Program, and 3) sharing information on how to sign up for the Program.

“A ROPS helps prevent injury or death in the event of an overturn. All Wisconsin farmers are eligible to apply for the rebate program, which reimburses up to 70 percent toward the total cost of purchasing, shipping, and installing individual ROPS,” (ROPS Rebate Program Staff; Wisconsin Ag Connection, May 2018).

In using this format, farmer quotes and stories were used; however, this was often done sparingly with little space provided for elaborate descriptions. In 2016; however, several media reports were published promoting the Program as a potential candidate for legislative funding. These media reports tended to be longer and included more detail about farmers’ experiences with tractor overturns as well as general safety issues. Only a handful of these media reports promoting the Program as a possible legislative effort were published in 2016, with one follow-up sharing an unfavorable legislative outcome in early 2018.

“This program makes Wisconsin farms safer. It’s very positive that the bill made it through both committees this year, but we’ll need at least one more big push to get it signed into law. This is something Wisconsin farmers need,” (Government Official; Wisconsin State Farmer, May 2018).

##### Media to introduce the ROPS Rebate Programs in unfunded states

Five media reports were published advocating for ROPS Rebate Programs in states not previously mentioned (Ohio, Indiana, Iowa, and Illinois). These media reports tended to share general information about the nationwide burden of tractor overturns and the need for a ROPS Rebate Program; however, little information was provided about next steps in establishing such a Program. Only one of these media reports was a follow-up to another.

## Discussion

The work carried out in this study provides evidence that media advocacy can be effective as a part of public health program implementation. In particular, honing in on correct and positive public health messages, the timing of media compared to that of the intervention, and the individuals involved in creating the media coverage (i.e. reporters and experts) were shown to influence the impact of media on implementation and sustainability of the ROPS Rebate Programs.

Across the different Programs, there were commonalities in how the Programs were portrayed that are likely to have had a positive impact, overall, on implementation and sustainability. Overall, the public health messages were positive, highlighting the Program as an expression of gratitude for farmers, the benefit of the Program to farmers and communities, and farmers’ interest in the Program. It was incredibly rare to find media reports with negative portrayals of the ROPS Rebate Program. Though true, it is also important to examine why so few negative media reports existed. First, the ROPS Rebate Programs are intended to save lives, a goal that was met and proven from early on [21]. As humans, it is ethical and socially expected to support any effort that saves lives. Despite this, the one negative portrayal of the ROPS Rebate Programs referenced the cost to the state as a reason not to implement the Program in Minnesota. However, a significant return-on-investment has been proven [14, 21] and used in the media to further elaborate on the Programs’ benefits.

Though the media reports were mainly positive, some information about the Programs was presented inaccurately. This raises the issue of ensuring that public health messages are clear and that, to whatever extent possible, all individuals speaking with the press are presenting consistent and accurate messages. Such confusion could be problematic for public health programs [22].

While the components of the public health messaging are important, in this study they were not sufficient for creating a meaningful impact on implementation and sustainment of the NRRP. Instead, it is important to examine the differences in media strategies (timing and spokespersons) used across the Programs. For the most part, media reports were published in response to ROPS Rebate Program launches; however, in Minnesota, media reports provided motivation for officials to launch a ROPS Rebate Program. Both of these strategies were beneficial; however, in Minnesota, the burden placed on Program staff and state champions to launch a ROPS Rebate Program was significantly reduced compared to that of other states. This can be important considering that limited project budgets and a wide array of competing priorities can limit the staff and champions’ ability to work on implementation efforts. In addition, in many cases funding is provided through government grants, meaning that that staff (including those working on the ROPS Rebate Programs) are not able to act as political advocates. Thus, media provides an alternative outlet for legislators and others to be educated about the benefits of public health programs.

In addition to being helpful prior to implementation, media advocacy after implementation can help sustain public health programs by keeping the program at the forefront. In New York, where media has been used regularly to highlight the impact of the ROPS Rebate Program, funding has been sustained for 12 years. Those states where media ended shortly after implementation tended to be the ones with less successful ROPS Rebate Programs (i.e. fewer rebate dollars and retrofits). Similarly, few stories have been published in Minnesota since the implementation of the Program. While funding was secured for a second year in a row, the amount was reduced and spread across 2 years rather than one. It is possible that the decreased media related to this issue created the illusion that tractor overturn fatalities are no longer an issue, and thus the Program is no longer needed. Further monitoring of media and legislative decisions over the next legislative session will provide additional insight into these trends and their relationship.

Given the importance of timing media related to public health programs, the question of generating enough interest to warrant media becomes problematic [22]. In Minnesota, the reporter who published the media report that motivated ROPS Rebate Program implementation, worked on his own without nudging from Program staff. However, in many cases, staff are instead required to pitch ideas to reporters. This becomes a challenge, as reporters may be faced with numerous priorities and interest [22]. Though not explicitly demonstrated by the results of this study, on-going working relationships between public health practitioners and media partners could be beneficial in disseminating media reports more easily. Even with an on-going relationship between public health practitioners and media partners, it is rare for a reporter to cover a story if that story is unlikely to generate interest within the public. In cases where the hook is not, on its own, sufficient for gaining the interest of the reporter, the individuals pitching the story may be of relevance.

This was the experience with the National ROPS Rebate Program launch, which was marginally covered by the press. However, a well-placed media report in POLITICO highlighted the financial struggles that the Program faced. A prominent US Senator with an interest in the Program then spurred tremendous media report in response to these issues as he worked to sustain Program funding. Similarly, in New York, legislators have played a key role in generating media about the ROPS Rebate Programs through both press conferences and personally authored media reports. During legislative sessions, the support of these politicians has been crucial for securing or restoring funding for the Program. Such media emphasizes the importance of gaining and maintaining political allies. In addition, the Programs make these alliances visible to the public, including farmers, who can then hold stakeholders accountable [23].

Though political support is extremely important for these Programs, the diversity of expertise (i.e. researchers, farmers, and legislators) presented in media reports examined in this study is also important for bringing the issue to the attention of diverse readers with a variety of interests. By presenting these diverse viewpoints, the media likely helped to further increase knowledge of, and demand and support for the Program, thus further encouraging legislative action.

In gaining traction, either at the policy level or within other groups, the source that media reports are published in can be of importance. Agricultural publications, which are directed primarily at the farm community, are useful in sharing information about the Program with the target population; however, these are less visible to policy makers and other supporters. Similarly, publications targeted at specific groups of stakeholders (for example, Occupational Safety and Health) can be useful in reaching individuals or organizations who wish to contribute to implementation. While these sources are important, the results of this study show the benefit of engaging policy makers in the discussion. The use of local, non-agricultural media sources (such as local newspapers), as well as more targeted media (e.g. POLITICO) can be useful in two ways. First, these sources have a greater potential to help capture the attention of policy makers [24]. Second, the sources provide a platform for policy makers to share their responses to the Program, as was seen primarily with the New York and National ROPS Rebate Programs.

Based on the results of this study, it is likely that the robustness of the media efforts surrounding the ROPS Rebate Programs can determine the trajectory of the Programs. In particular, media reports that feature a variety of expertise are present in states with strong financial support for the ROPS Rebate Programs. These varied expert views of the ROPS Rebate Program provide ample opportunity for the benefits of the Program to be highlighted in several ways, making it easier to appeal to supporters with varying agendas and views.

Moving forward, robust media providing diverse perspectives on the many benefits of the ROPS Rebate Programs could be used as viable implementation strategies as additional states look to launch ROPS Rebate Programs. Ideally, local champions and reporters would work together to provide adequate coverage to begin a “domino effect” similar to what was seen in Minnesota. Once launched, these same collaborations can be used to further highlight the local impact of the Program and engage policy-makers and supporters in public discussions of the Program. By making both the implementation and sustainment of the Program public through use of media, the commitments made by various parties become part of an accountability plan which encourages these partners to continue their support, thus promoting the success of the Programs.

### Strengths and limitations

This study has two main limitations. First, some relevant media reports may not have been captured at the time of publication or available online at the time of this study. Thus, it is possible that important sources of information were missed. Second, it was outside of the scope of this study to interview the journalists and Program supporters referenced in the media reports for their perspectives on publicly supporting the ROPS Rebate Program. The knowledge that could be generated from such discussions, particularly related to motivation for their involvement in media reports, would be helpful in understanding the relationship between media and the trajectory of the ROPS Rebate Programs. Such interviews could be the basis of a follow-up study.

A strength of this study is the trustworthiness of the analysis. Both the first and second authors have been heavily involved in these Programs, and thus, are biased to certain aspects of the media. To reduce this bias and improve trustworthiness of the study, several individuals with different levels of involvement in the Programs were involved in the analysis. This included two authors who are heavily involved in Program administration, two who have primarily been involved in evaluation of the Program, and one external reviewer who had not been exposed to the ROPS Rebate Programs ahead of this study. Though there were no disagreements related to content between the authors’ analysis and that of the external reviewer, the two summaries placed different emphases on different aspects of the media reports. In the results, these viewpoints were combined to reduce any relevant biases.

## Conclusions

The use of media to implement and sustain the ROPS Rebate Programs has been documented across the different Programs. Those Programs which used more comprehensive media strategies also are those with more promising funding structures and thus greater overall success. Though it can be difficult to distinguish which came first – ample media or overall Program success - this suggests that media advocacy can be an important component of implementation strategies, not just in occupational safety settings, but in public health overall.

Such strategies have the potential to not only influence relevant policy-makers, but also require accountability by program supporters who make their commitments public. By identifying appropriate media champions and utilizing media before, during, and after implementation, public health programs can expect to see benefits.

## Data Availability

A database of the media articles used in this study is maintained by National ROPS Rebate Program staff and may be obtained by contacting Pamela Tinc (pam.tinc@bassett.org).

## Abbreviation

ROPS: Rollover protective structures
